# Allostatic load index across the psychosis spectrum: a systematic review and meta-analysis

**DOI:** 10.3389/fpsyt.2025.1590547

**Published:** 2025-07-01

**Authors:** Lander Madaria, Claudia Aymerich, Borja Pedruzo, Gonzalo Salazar de Pablo, Daniel Alonso-Alconada, Paolo Fusar-Poli, Miguel Ángel Gonzalez-Torres, Ana Catalan

**Affiliations:** ^1^ Psychiatry Department, Basurto University Hospital., Bilbao, Spain; ^2^ Biobizkaia Health Research Institute, Organización Sanitaria Integrada (OSI) Bilbao-Basurto, Bilbao, Spain; ^3^ Neuroscience Department, University of the Basque Country, UPV/EHU, Leioa, Spain; ^4^ Centro de Investigación en Red de Salud Mental (CIBERSAM), Madrid, Spain; ^5^ Department of Child and Adolescent Psychiatry, Institute of Psychiatry, Psychology & Neuroscience (IoPPN), King’s College London, London, United Kingdom; ^6^ Child and Adolescent Mental Health Services, South London and Maudsley NHS Foundation Trust, London, United Kingdom; ^7^ Department of Child and Adolescent Psychiatry, Institute of Psychiatry and Mental Health. Hospital General Universitario Gregorio Marañón School of Medicine, Universidad Complutense, IiSGM, CIBERSAM, Madrid, Spain; ^8^ Department of Cell Biology and Histology, School of Medicine and Nursing, University of the Basque Country (UPV/EHU), Leioa, Spain; ^9^ Early Psychosis – Interventions and Clinical-detection (EPIC) Lab, Department of Psychosis Studies, Institute of Psychiatry, Psychology and Neuroscience, King's College London, London, United Kingdom; ^10^ Department of Brain and Behavioral Sciences, University of Pavia, Pavia, Italy. Outreach and Support in South-London (OASIS) Service, South London and Maudsley (SLaM) NHS Foundation Trust, London, United Kingdom; ^11^ Department of Psychiatry and Psychotherapy, University Hospital, Ludwig-Maximilian-University (LMU), Munich, Germany

**Keywords:** allostatic load, psychosis, schizophrenia spectrum disorders, first-episode psychosis, allostatic load index

## Abstract

**Background:**

Individuals diagnosed with schizophrenia spectrum disorders experience significantly higher morbidity and mortality rates than the general population, with evidence of multisystemic alterations. The concept of allostatic load (AL) provides a framework for understanding the cumulative physiological burden imposed by chronic stress. This burden is quantified using the AL index, which integrates multiple biomarkers to assess the impact of prolonged stress on various physiological systems. This review aims to measure the difference in the AL index between individuals with psychosis and the general population, as well as to evaluate the methods used to assess AL in this population.

**Methods:**

A PRISMA/MOOSE-compliant systematic search was conducted in the Web of Science, PubMed, BIOSIS, KCI-Korean Journal Database, MEDLINE, Russian Science Citation Index, SciELO, and Cochrane Central Register databases from inception to January 28th, 2025. Studies reporting on the AL index of individuals with psychosis or clinical high risk of psychosis (CHR-P) compared to healthy controls (HC) were included. We used random effects meta-analysis to evaluate: (1) differences between patients with a chronic schizophrenia spectrum disorder (C-SSD) or first-episode psychosis (FEP), compared to healthy controls (HC); (2) differences between patients with C-SSD and FEP. We conducted quality assessment, heterogeneity, publication bias, and meta-regression analyses (PROSPERO: CRD 42024579704).

**Results:**

From 922 citations, five studies were included (N=669), showing a higher AL in individuals with psychosis (C-SSD, k=3; g= 1.3315; 95% CI: 0.9679–1.6951; FEP, k=4; g = 0.5464; 95% CI, 0.0698 to 1.0230) compared to HC. The AL index was also higher in patients with C-SSD compared to FEP (k=3; g = 0.8196; 95% CI, 0.2977 to 1.3415). No CHR-P data were found for analysis. Different methods for computing the AL index were observed.

**Conclusion:**

Allostatic load seems higher in individuals with psychosis compared to the general population, with chronic conditions exhibiting higher allostatic load than the early stages of the disorder. However future research is needed to consolidate these emerging trends.

## 1 Introduction

The lifetime prevalence of psychotic disorders is estimated to be over 3% (1), resulting in a substantial economic, social (2) and subjective burden (3). Schizophrenia, once primarily regarded as a disorder of central nervous system dysfunction, has increasingly been investigated from a multisystemic perspective in recent years (4). Emerging evidence suggests that alterations in antipsychotic-naïve, first-episode psychosis (FEP) individuals, extend across multiple systems, including metabolic (5), neuroendocrine (6), and immunological alterations (7, 8). Additionally, individuals diagnosed with schizophrenia tend to have poorer dietary habits (9, 10), lead more sedentary lifestyles (11) and exhibit higher smoking rates (12). The well-established association between antipsychotic treatment and its metabolic side effects further exacerbates the decline in overall health (13, 14). Consequently, this population experiences worse physical health (15) and reduced life expectancy compared to the general population (16), with this gap steadily increasing over time (17).

In this context, the role of stress as a mediating factor in psychosis, along with its physiological effects, has gathered increasing attention (18–20). Certain extreme psychosocial stressors, such as childhood traumatic events, have been linked to psychotic disorders in adulthood (21, 22). Additionally, greater exposure to psychosocial stress, emotional abuse, and perceived discrimination has been shown to significantly heighten the risk of transition to psychosis in individuals at clinical high risk for psychosis (CHR-P) (18). Various models have been proposed to investigate the relationship between stress and psychosis, including the adaptive calibration model (23), the reactive scope model and the neural diathesis-stress model (24). More recently, the concept of allostatic load (AL) has been suggested as a useful paradigm, as it provides a measurable construct—the AL index—which encompasses multiple biological markers altered by chronic stress (25).

AL, first defined by McEwen and Stellar (26) in 1993, is a concept that explains the physiological consequences caused when an organism’s adaptive responses to stressors become maladaptive, also defined as the “wear and tear” exacted on the organism by chronic stress (27).

To quantify AL, a set of stress-associated biomarkers that undergo sustained alterations over time has been employed (28). However, a recent research indicates that, there is still no consensus on the specific components required for the formulation of the AL index (29). Most studies employ metabolic, cardiovascular, neuroendocrine, and immunological parameters, as these tend to undergo lasting changes following chronic stress exposure (30).

The most used methodology for calculating the AL index involves distributing each parameter’s values into quartiles and assigns a score of 1 to those parameters that fall into the quartile closest to altered values. Thus, parameters such as blood pressure would receive a point if they are in the upper quartile, while parameters like high density lipoprotein (HDL) or dehydroepiandrosterone (DHEA) values in the lower quartile would also score 1 point (31).

However, the reference values used for this classification have varied across studies. Some studies have utilized healthy controls from their own sample (32), while others have relied on predefined standard reference values (30). In cases where a control group was not available, the only existing cohort within the study has been used as the reference (31).

Regarding the calculation of the AL index, some authors have suggested summing all the assigned points (30), whereas others advocate for grouping the parameters by categories, such as the neuroendocrine or metabolic system, calculating an average for each group, and balancing the weight of each system in the final sum (33).

The concept of AL has been linked to an increased prevalence of diseases and a higher risk of mortality in old age (34, 35). In mental health, it has been also studied as a mediator between traumatic experiences and depressive symptoms (36), as well as being associated with anxiety, depression, and suicidal symptoms (37). It is also a factor related to functioning and hyperreactivity in bipolar disorder (38). When it comes to psychosis, research on AL is rapidly expanding, showing higher levels in individuals with psychosis and being associated with higher severity of positive and depressives symptoms and lower overall functioning (39).

To date, the evidence on AL in psychosis relies exclusively on independent observational studies, without any integrated data analysis among them. Furthermore, findings are often inconclusive due to small sample sizes and the heterogeneity of the included samples. Our aim is to study the association of AL at different stages of psychotic disorders compared with the general population, and to assess with the moderating effect of variables such as sex, age, symptom severity and study quality. Furthermore, we seek to evaluate the consistency of the methods used to measure AL index across studies analyzing this parameter within the psychosis spectrum.

## 2 Methods

This study was registered in PROSPERO (CRD42024579704). This systematic review and meta-analysis were conducted according to the PRISMA 2020 (**Supplementary Table S1**) (40) and the MOOSE checklists (41) (**Supplementary Table S2**), following the EQUATOR Reporting Guidelines (42).

### 2.1 Search strategy and selection criteria

A systematic search strategy was used to identify relevant articles, and two-step literature search was implemented by two independent researchers (LM, CA). The Web of Science database (Clarivate Analytics) was searched, incorporating the Web of Science Core Collection, BIOSIS Citation Index, KCI-Korean Journal Database, MEDLINE, Russian Science Citation Index, and SciELO Citation Index, as well as the Cochrane Central Register of Reviews and Ovid/PsycINFO databases. The search was conducted in English from inception until January 28, 2025. The following search terms were applied: ((ultra-high risk OR clinical high risk OR BLIPS OR prodrom* AND psychosis OR psychotic) OR schizophren* OR psychosis OR psychot*) AND (allosta* OR alosta*). Articles identified through this search were screened at the abstract level. After excluding those that did not meet the inclusion criteria, the full texts of the remaining articles were assessed for eligibility, and decisions were made regarding their inclusion in the review.

The PICOS framework was used to define eligibility criteria, focusing on observational studies comparing allostatic load between healthy controls, individuals with FEP, and those with chronic schizophrenia spectrum disorders (C-SSD).

Thus, inclusion criteria were the following: (1) individual studies presenting original data; (2) reporting on a sample of patients meeting criteria for clinical high-risk (CHR-P) defined according to established psychometric instruments (e.g., CAARMS (43) or SIPS (44)), FEP (defined as patients presenting with psychosis under 5 years from onset), or a schizophrenia spectrum disorder, according to ICD (45) or DSM (46) criteria; (3) including a HC comparison group; and (4) providing quantitative data on the AL index, as defined by the study authors. No language restrictions were applied. Exclusion criteria were (1) studies focusing on patients with affective psychotic disorders, (2) studies lacking a control group, and (3) overlapping samples. Overlap was assessed by analyzing the studies inclusion dates, the type of population studied, and the country where the research was conducted. In case of overlapping, we chose either the largest sample or the study that includes the most subgroups among those considered.

### 2.2 Outcome measures and data extraction

Two researchers (LM and CA) independently extracted data from all included studies into the database, that was later cross-checked. When there were doubts about whether to include an article or how to extract its relevant information, a senior researcher (AC) made the final decision. The summary of included variables comprised the following information: first author and year of publication, site, recruiting period and follow-ups, study design, sample size, sample type (C-SSD, FEP or CHR-P), diagnostic criteria, age (mean and standard deviation [SD]), sex, education, allostatic load index in each group, AL index definition and components, clinical outcomes (measured by Positive and Negative Syndrome Scale (PANSS) (47)), quality assessment (according to the Newcastle Otawa scale (NOS), see below (48)), and key findings. In accordance with the inclusion criteria of the original studies, we used the term C-SSD to refer to individuals with chronic conditions within the schizophrenia spectrum. The main outcome, AL index, was extracted as defined by the authors. To reduce the amount of missing data, we utilized WebPlotDigitizer version 5.2 (49) to extract information available solely from figures (50).

### 2.3 Strategy for data analysis

Anticipating high heterogeneity among studies, random-effects meta-analyses were performed (51). Heterogeneity was evaluated using the Q statistic, and the proportion of variability attributable to heterogeneity was quantified with the I² index (52). Potential publication bias was assessed through visual inspection of funnel plots and statistical testing using Egger’s test (53).

First, the standardized mean difference (Hedges’ g) was calculated for each study using the reported means and standard deviations. Then, Hedges’ g values from the different studies were pooled in a meta-analysis for each of the available comparisons: FEP vs HC, C-SSD vs HC, FEP vs C-SSD, and C-SSD (including both FEP and chronic schizophrenia samples) vs HC.

Despite of the limited number of studies, we conducted meta-regression analyses to estimate the association between AL index and outcomes to estimate the association between the AL index and the (1) mean age, (2) sex (% females), (3) psychosis severity (using the PANSS scale (47)), and (4) quality of the study (total NOS score).

In order to evaluate the consistency of the AL index measurement criteria across the five analyzed studies, a frequency analysis of each employed biomarker was conducted.

### 2.4 Risk of bias (quality) assessment

For study appraisal, we used the NOS (48), which assesses study quality across three domains: selection, comparability, and outcome. The selection domain includes four categories evaluating sample representativeness, sample size, non-response rate, and exposure ascertainment, with a maximum of five stars. The comparability domain assesses control for confounding factors, allowing up to two stars. The outcome domain consists of two categories evaluating outcome measurement and the appropriateness of statistical tests, with a maximum of three stars. Based on the total number of stars awarded, studies are classified as good, fair, or poor quality.

## 3 Results

**Figure 1:**
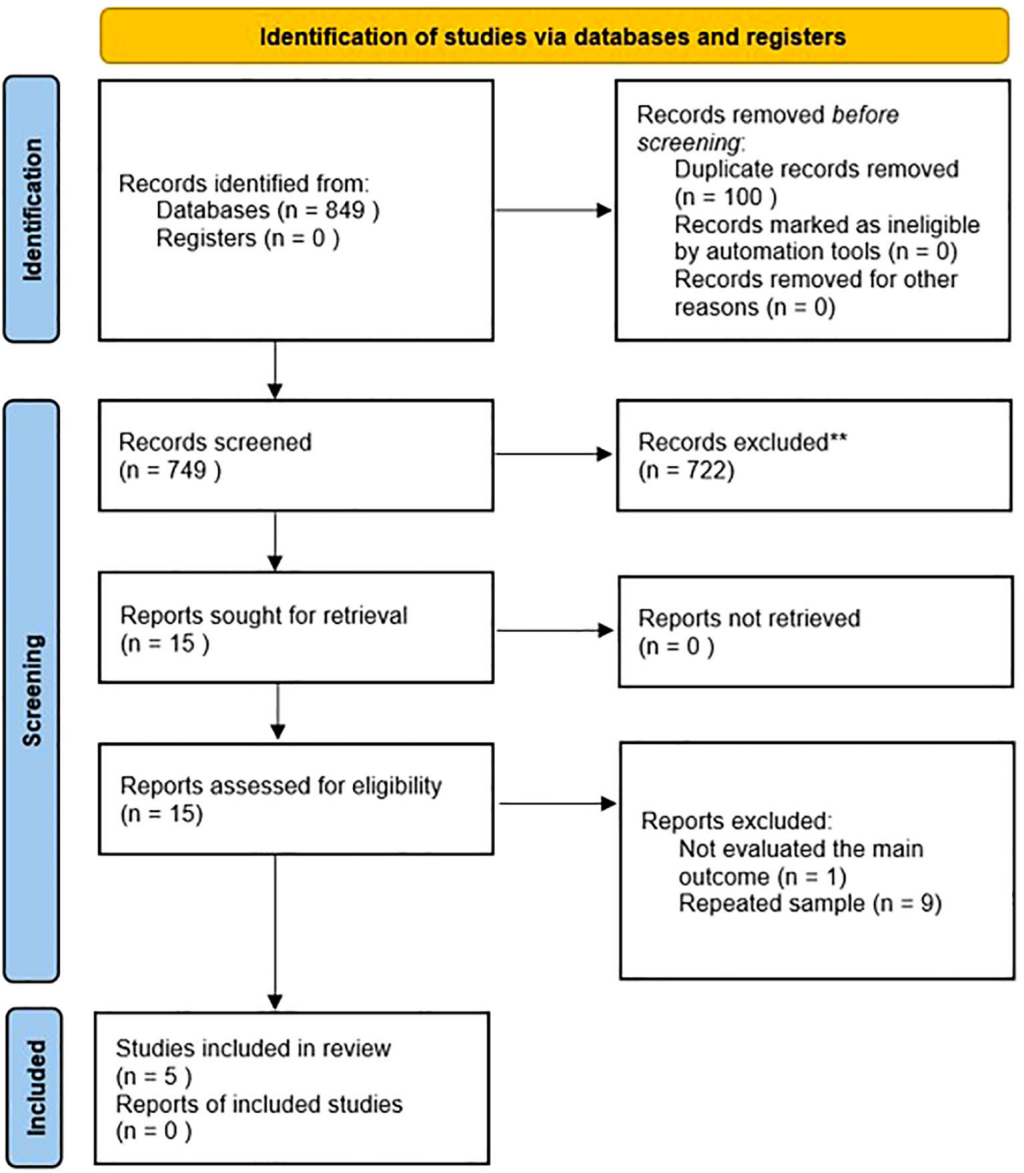
PRISMA flowchart.

The literature search yielded 922 citations, which were screened; 15 full-text articles were assessed for eligibility. After excluding those not meeting the inclusion criteria, 5 studies were included (54–58), reporting on 4 independent cohorts (**Figure 1**).

As shown in **Table 1**, four studies provided data on individuals diagnosed with psychosis (54–57), three on individuals with C-SSD (54–56), and four on individuals with FEP (54–56, 58). Only one of the studies found included data on CHR-P individuals (59), but it was excluded from the analysis due to the lack of a control group.

**Table 1:** characteristics of included studies.

Author & year	Nos	Patients		Controls		Sample type	Diagnosis method
		n (M/F)	Age (mean ± SD)	n (M/F)	Age (mean ± SD)		
Piotrowski2019 (55)	9	CHRONIC 25 (14/11) FEP 42 (21/21)	CHRONIC 48,8 ± 13,8 FEP 27,7 ± 7,3	42 (16/26)	27,8 ± 8,4	FEP, SCZ-AR, FHR-P	OPCRIT
Berger2018 (54)	8	CHRONIC 28 (19/9) FEP 28 (15/13)	CHRONIC 40,07 ± 10,12 FEP 32,96 ± 11,49	53 (36/17)	36,34 ± 11,49	FEP, SCZ	SCID-IV
Savranski2018 (56)	9	CHRONIC 37 (25/12) FEP 21 (16/5)	CHRONIC 43,35 ± 12,91 FEP 23,41 ± 4,34	34 (20/14)	35,26 ± 14,03	FEP, SCZ	SCID-IV and V (More than 5 years since onset)
Zhou2021 (57)	9	PSYCHOSIS 167 (93/74)	CHRONIC 37,59 ± 13,78	72 (36/36)	39,25 ± 12,01	SCZ	SCID-IV
Zhou2020 (58)	9	FEP 79 (38/41)	FEP 27,2 ± 7,6	41 (21/20)	29,8 ± 6,4	FEP	SCID-IV (First episode schizophrenia within 2 weeks of treatment)
Total		427 (241/186) CHRONIC 257 (151/106) FEP 170 (90/80)	34,11 ± 12,96 CHRONIC 39,78 ± 13,12 FEP 27,80 ± 7,98	242 (129/113)	34,45 ± 11,05		

FEP, first-episode psychosis; FHR-P, individuals at familial high risk of psychosis; OPCRIT, the Operational Criteria for Psychotic Illness Checklist[87] [87]; SCID, the Structured Clinical Interview for DSM-IV or V[45]; SCZ, schizophrenia; SCZ-AR, schizophrenia acute relapse;

The overall database comprised 549 individuals, with 348 diagnosed with psychosis (41.67% women, 36.8 ± 12.5 years). 90 were diagnosed with C-SSD (35.56%% women, 43.84 ± 12.82years), 170 with FEP (47.06% women, 27.8 ± 7.98 years), and 242 were HC (46.69% women, 34.45 ± 11.05 years).

### 3.1 AL index in subjects with psychosis vs HC

**Figure 2:**
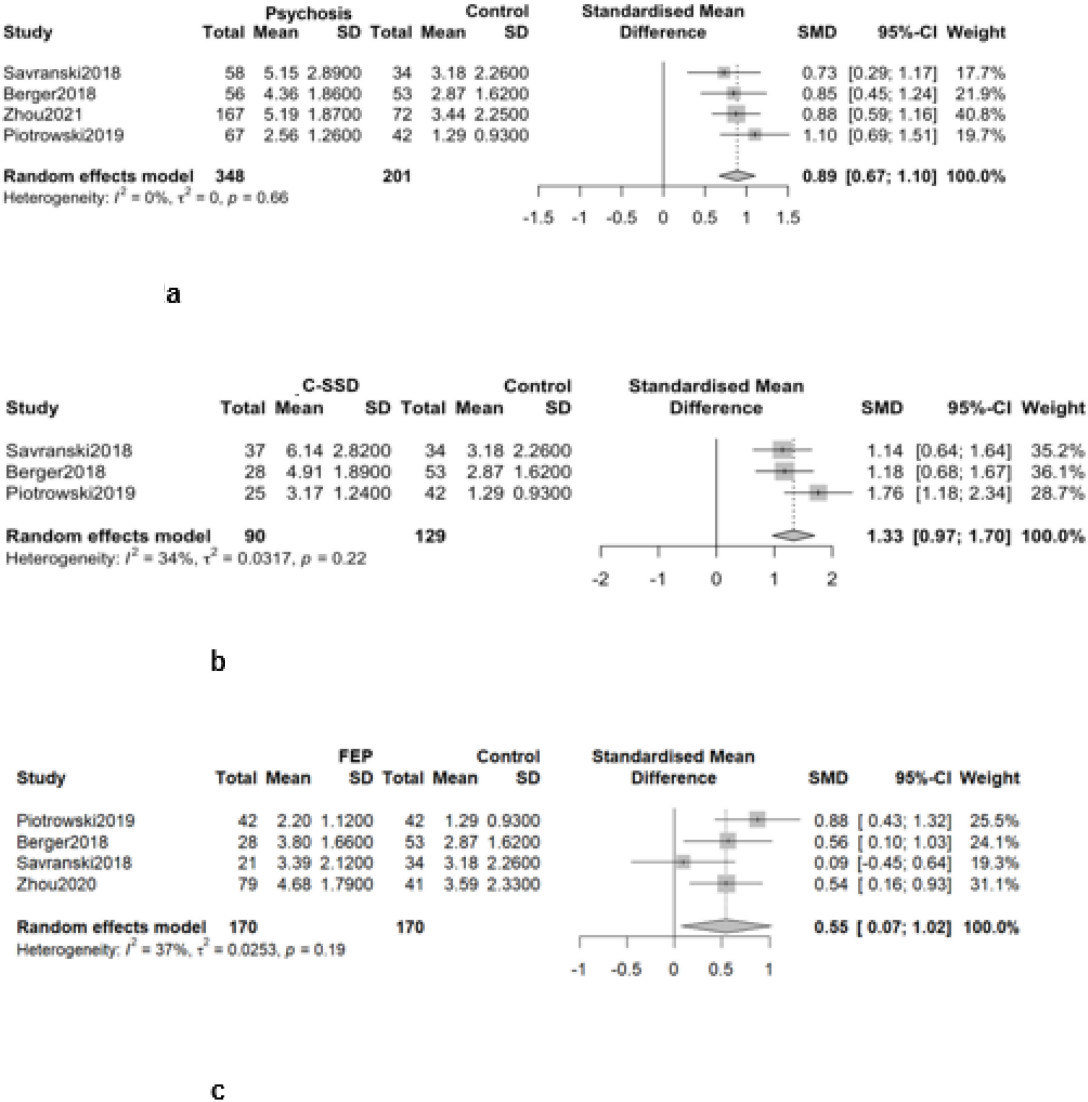
Comparison of AL index between psychoses groups and HC. **(a)** Comparison of AL index between psychosis and control. **(b)** Comparison of AL index between C-SSD and control. **(c)** Comparison of AL index between FEP and control.

When comparing AL index between individuals with psychosis with healthy controls samples, the analysis revealed a significantly higher AL index in the psychosis group (**Figure 2a**) (k=4; g = 0.8882; 95% CI: 0.6722–1.1043). A similar pattern was observed when comparing individuals with C-SSD to the HC group (**Figure 2b**), with a markedly higher AL index (k=3; g = 1.3315; 95% CI: 0.9679–1.6951). Likewise, in the FEP group (**Figure 2c**), AL index was significantly elevated compared to HC (k=4; g = 0.5464; 95% CI: 0.0698–1.0230).

**Figure 3:**
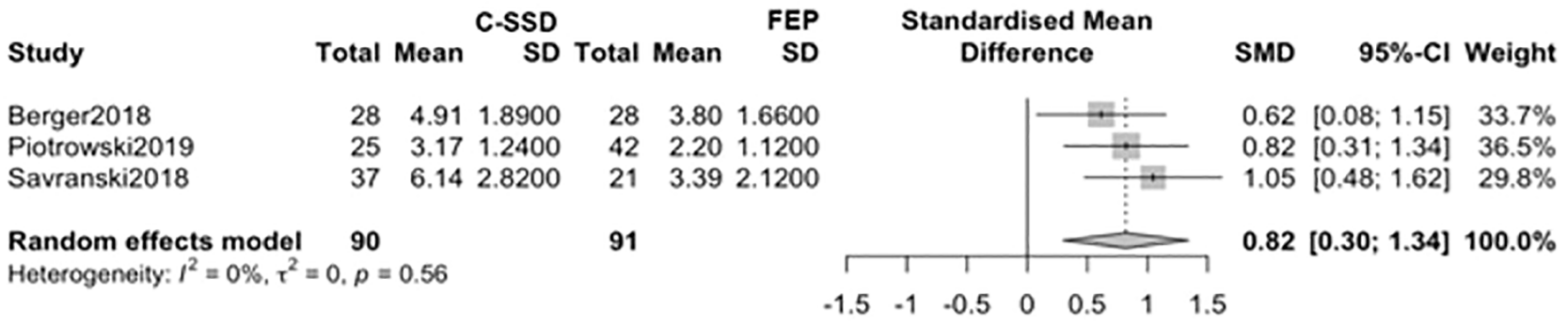
Comparison of AL index between C-SSD and FEP groups.

Furthermore, a statistically significant difference was found when comparing the C-SSD group to the FEP group (**Figure 3**), indicating a higher AL index in chronic patients (k=3; g = 0.8196; 95% CI: 0.2977–1.3415).

### 3.2 AL index measurement system

**Figure 4:**
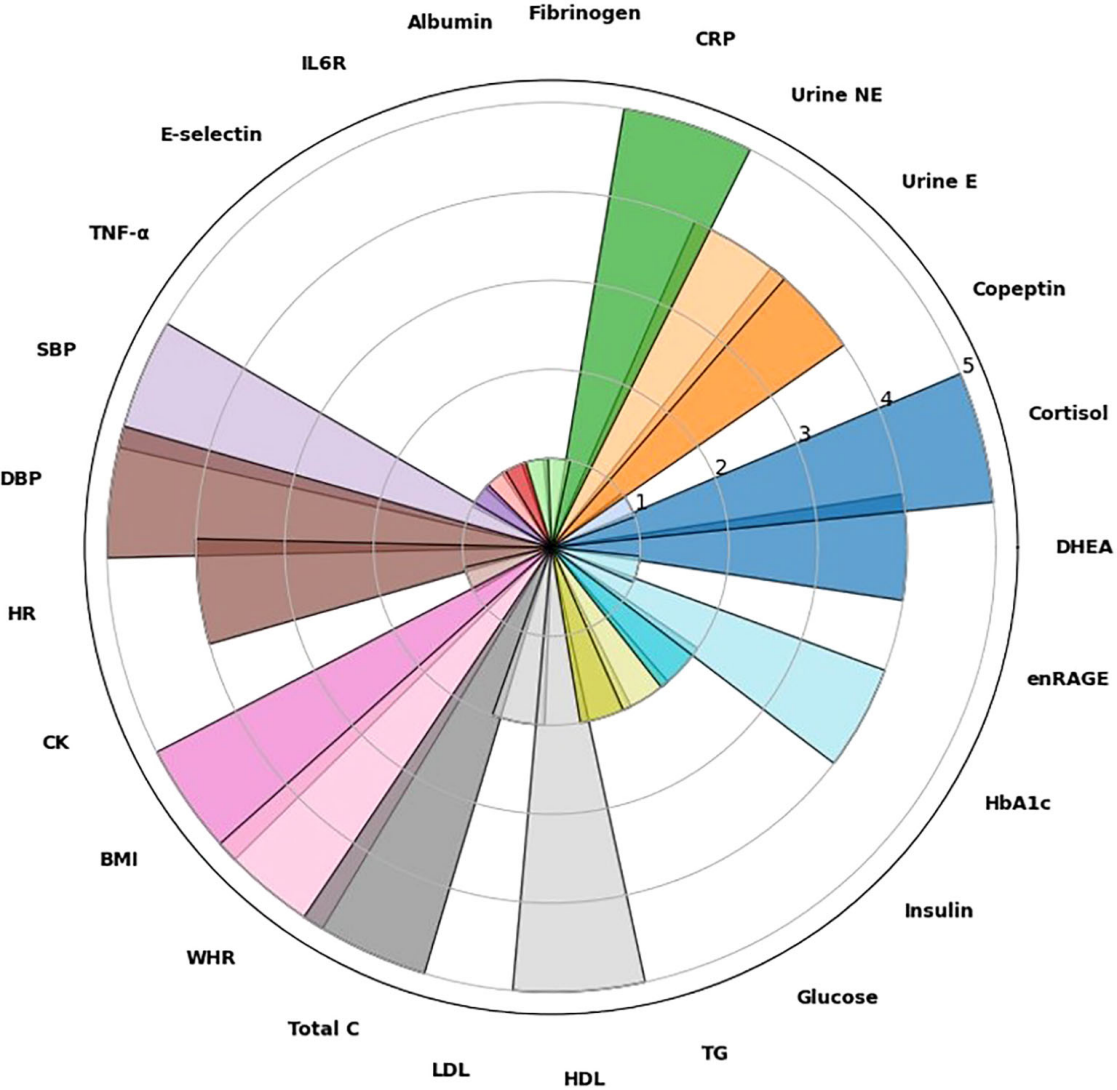
Frequency of biomarkers included in AL scores across all 5 studies retained for the systematic review. BMI, body mass index; CK, creatinine kinase; CRP, C-reactive protein; DBP, diastolic blood pressure; DHEA, dehydroepiandrosterone; enRAGE, extracellular newly identified RAGE-binding protein; HbA1c, glycosylated hemoglobin; HDL, high-density lipoproteins; HR, hearth rate; IL6R, interleukin 6 receptor; LDL, low-density lipoproteins; SBP, systolic blood pressure; TC, total cholesterol; TG, triglycerides; TNF-a, tumor necrosis factor-a; UriEph, urine epinephrine; UriNEph, urine norepinephrine; WHR, waist-to-hip ratio.

To evaluate the consistency of the AL index measurement criteria across the five meta-analyzed studies, the frequency of use of each biomarker was recorded and distributed (**Figure 4**) as follows: 8 biomarkers (cortisol, C-reactive protein [CRP], systolic blood pressure [SBP], diastolic blood pressure [DBP], body mass index [BMI], waist-to-hip ratio [WHR], total cholesterol [Total C], and high-density lipoprotein [HDL]) were used in all five studies. 5 biomarkers (dehydroepiandrosterone [DHEA], urinary epinephrine [Urine E], urinary norepinephrine [Urine NE], heart rate [HR], and glycated hemoglobin [HbA1c]) were reported in four studies. 4 biomarkers (low-density lipoprotein [LDL], triglycerides [TG], glucose, and insulin) were reported in two studies, and finally, 8 biomarkers (copeptin, fibrinogen, albumin, interleukin-6 receptor [IL6R], E-selectin, Tumor Necrosis Factor-alpha [TNF-α], creatine kinase [CK], and extracellular newly identified RAGE-binding protein [enRAGE]) were only reported in one study.

Some biomarkers, such as DHEA, were measured differently, with one study opting to analyze its sulfated form (55). Others, like cortisol, were obtained from different biological samples depending on the study, while epinephrine and norepinephrine were measured through their metabolites, metanephrine and normetanephrine, respectively, in one study (54).

As shown in **Table 2**, when calculating the AL index, some biomarkers were grouped differently depending on the study methodology. While all studies use the reference control group biomarker values to calculate risk quartiles, only two of them calculate sex specific cut-offs (54, 55).

**Table 2:** AL index measurement.

Autohr & year	Biomarkers and categories	Computation	Range
Piotrowski2019 (55)	CV: SBP, DBP ANT: BMI, WHR INFL: hsCRP, fibrinogen, albumin GLU: glucose, insulin LIP: TC, LDL, HDL, TG STEROIDS: cortisol, DHEA-S	Based on the sample’s distribution of biomarker values. Divided by categories: Sex specific cut-off calculated	0 to 6
Berger2018 (54)	CV: SBP, DBP, HR, CK NE: cortisol, copeptine, metanephrine, normetanephrine INM: TNF-a, IL6R, CRP, eselectin MET: BMI, WHR, insulin, glucose, HbA1c, enRAGE, TG, TC, LDL, HDL,	Based on the sample’s distribution of biomarker values. Divided by categories: Sex specific cut-off calculated	0 to 4
Savranski2018 (56)	CV: SBP, DBP HR MET: BMI, WHR, HDL, TC, HbA1c INF: CRP Stress: UrEph, UrNeph, Ucor, DHEA	Based on the sample’s distribution of biomarker values. Not divided by categories: Not sex specific cut-off calculated	0 to 13
Zhou2021 (57)	CV: SBP, DBP, HR; MET: BMI, WHR, HDL,TC,HbA1c; INF: hCRP; NE: UriEph, UriNEph, UriCor, DHEA	Based on the sample’s distribution of biomarker values. Not divided by categories: Not sex specific cut-off calculated	0 to 13
Zhou2020 (58)	CV: SBP, DBP, HR; MET: BMI, WHR, HDL,TC,HbA1c; INF: hCRP; NE: UriEph, UriNEph, UriCor, DHEA	Based on the sample’s distribution of biomarker values. Not divided by categories: Not sex specific cut-off calculated	0 to 13

ANT, anthropometric; BMI, body mass index; CK, creatinine kinase; CRP, C-reactive protein; hsCRP, high sensitive C-reactive protein; DBP, diastolic blood pressure; CV, cardiovascular; DHEA, dehydroepiandrosterone; DHEA-S, dehydroepiandrosterone sulfate; enRAGE, extracellular newly identified RAGE-binding protein; GLU, glucose metabolism; HbA1c, glycosylated hemoglobin; HDL, high-density lipoproteins; HR, hearth rate; IL6R, interleukin 6 receptor; INF, inflammatory; LDL, low-density lipoproteins; LIP, lipid metabolism; MET, metabolic; NE, neuroendocrine; SBP, systolic blood pressure; TC, total cholesterol; TG, triglycerides; TNF-a, tumor necrosis factor-a; UriEph, urine epinephrine; UriNEph, urine norepinephrine; UriCor, urine cortisol; WHR, waist-to-hip ratio.

### 3.3 Metaregressions

The meta-regression analyses conducted for age, sex, and study quality did not find statistically significant differences (p>0.05). Similarly, no significant associations were identified when exploring the potential relationship between AL index and symptom severity through meta-regression analyses for the PANSS-N, PANSS-P, and PANSS-G scales (47), in individuals diagnosed with C-SSD, FEP, or in the general psychosis analysis. Detailed information is available in **Supplementary Table S4**.

### 3.4 Heterogeneity and publication bias assessment

Heterogeneity varied across comparisons, ranging from 0.0% to 36.6%. Moderate heterogeneity was observed in the C-SSD vs. control group (*I² = 59.4%*, *Q* = 7.39, *p* = 0.0606) and in the FEP vs. control group (*I² = 36.6%*, *Q* = 4,73, *p* = 0.1928) while the FEP vs. C-SSD comparison showed no significant heterogeneity (*I² = 0.0%*, *Q* = 1.16, *p* = 0.5585) as well as psychosis vs. control group (*I² = 0.0%*, *Q* = 1.58, *p* = 0.6647). Publication bias was not identified through visual inspection of funnel plots (Supplementary figure S1) for neither of the studied comparisons, and no statistical tests for small-study effects were conducted due to the limited number of studies in each comparison.

## 4 Discussion

To the best of the authors’ knowledge, this is the first meta-analysis to examine whether individuals with psychosis exhibit significant differences in terms of AL compared with HC. Our study indicates that the AL index is higher in individuals with psychosis spectrum diagnosis compared to healthy controls. Furthermore, individuals with chronic schizophrenia exhibit higher AL index than those in early stages of the illness. This supports the theory that AL reflects multisystem deterioration driven by chronic stress (26). However, these findings are based on a small dataset and require further consolidation.

There are several potential explanations for these findings. First, individuals with chronic conditions tend to be older, and aging itself is a factor associated with an increased AL (60). Also, individuals diagnosed with schizophrenia spectrum disorders often face worse social determinants of health (61), which are linked to elevated allostatic load (62), along with a high prevalence of unhealthy lifestyle factors (63, 64). Another important aspect to consider is the relationship between AL biomarkers and metabolic syndrome. Systolic blood pressure, diastolic blood pressure, HDL, triglycerides, and waist circumference comprise the values that determine metabolic syndrome (65), and, as previously stated (30), they are used in the computation of the AL index. On the other hand, it is well known that pharmacological treatment itself significantly alters these parameters (13, 66), although a higher prevalence has also been observed among drug-naïve individuals (67). It has been also observed that key stress-related biomarkers, such as cortisol (6), norepinephrine (68), and DHEA (69), as well as cytokines (70) and C-reactive protein (71), show individual alterations in psychosis. These findings support the allostatic load (AL) measurements, as they reflect the same physiological dysregulation associated with chronic stress in this population.

The role of psychosocial stress associated with a psychosis diagnosis should not be overlooked. The social stigma, associated with individuals with mental disorders (72), could be considered another determining factor for an increased AL. Notably, similar results have been observed in other groups facing social disadvantages, including individuals from racially and ethnically diverse backgrounds (73) and members of minority communities (74).

Alternatively, the psychotic symptoms themselves should be regarded as a stressor. This study did not find a statistically significant relationship between a higher AL index and greater symptom severity in psychosis, probably due to very limited statistical power. However, the work of Piotrowsky et al. (55) reported such a relationship. Moreover, other included studies (54, 56–58) have reported a correlation between a higher AL index and positive symptom subscales. Additionally, Berger et al. (54) examined AL index during acute psychotic episodes and after the initiation of treatment, observing a reduction in AL following psychopathological stabilization. This finding is consistent with studies linking elevated AL to worse problem-solving coping strategies and increased depressive symptoms (75, 76). However, some studies did not find a significant relationship between AL and self-appraisal of stress (55, 76). As a physiological marker, AL differs from perceived stress, which reflects a subjective interpretation of emotional tension. This distinction suggests that physiological and perceived stress may involve different underlying mechanisms.

It is also important to note that early life stress play a significant role in the genesis of the psychotic disorder (21, 22). This relates with AL, both in general population (77) and individuals with psychosis connecting an elevated AL index and events such as sexual abuse and parental antipathy (78). In our review, one study, excluded from the quantitative analysis, examined the AL index in individuals with CHR-P (59), found a correlation with poorer social and occupational functioning, as well as mania symptomatology. Although other studies have reported altered stress-related biomarker levels in this population (20, 79), it is important to note that, to date, no study has compared the AL index of CHR-P individuals with a control group. On the other hand, other studie (55) evaluated the AL index in relatives of patients with schizophrenia, finding that they exhibit a higher AL than HC and a similar level to individuals with FEP.

Neuroanatomical studies have also demonstrated a relationship between elevated AL and structural changes, including alterations of the fornix connectivity (80), reduction in prefrontal cortex thickness (81) and elongation of the choroid plexuses (58). AL has been associated with reduced brain plasticity (82), which, in turn, has been linked to impaired global cognition and executive function, with no significant impact on memory (83). The association between the brain plasticity and the cognition has been previously documented in the literature (84) and this may also explain the observed association between AL and greater cognitive impairment in psychosis (57, 85).

Nevertheless, considerable heterogeneity exists in both the biomarkers used to determine the AL index and the methods for its computation. While all analyzed studies align with Juster et al.’s (86) definition of the “Group Allostatic Load Index” when using reference control values, differences arise in category selection, such as sex adjustments, a variation noted in other studies (38). Despite similar findings across computational approaches (33), a recent meta-analysis suggests that an AL index including C-reactive protein, resting heart rate, HDL, waist-to-hip ratio, and glycosylated hemoglobin may better predict adverse health outcomes (28), though its exclusion of HPA-axis markers weakens its connection to stress as a causal factor. Integrating the AL index is crucial, as it can serve as a prognostic tool in clinical practice and promote a multisystemic approach to psychosis, applicable from early detection to chronic stages and even preventive strategies. Future integration into clinical frameworks will require validation of its sensitivity, specificity, and utility in real-world settings.

It is essential to move toward the development of a unified allostatic load model, supported by a standardized and consensual computational procedure that enables its application in broad, homogeneous, and methodologically robust analyses. Implementing this model from a transdiagnostic perspective would allow for a deeper understanding of the symptoms and phenomena most closely associated with allostatic load in diverse populations, thereby enhancing our comprehension of its underlying mechanisms and clinical relevance. Moreover, this approach would also facilitate a better understanding of the relationship between allostatic load and potential confounding variables, such as substance use, antipsychotic medication, sex, or other relevant factors.

This study must be interpreted within its limitations. One of the main limitations of the study is the small sample size, with only five studies included, comprising four different cohorts. This has made it impossible to assess the relationship between AL and relevant aspects such as antipsychotic medications, tobacco and alcohol use, or other illicit substances. On the other hand, the previously mentioned variability in the computation of the AL index represents a limitation; however, we sought to address this by focusing on relative differences in allostatic load across groups. Additionally, another aspect to consider is that, in one of the studies (54), the FEP group consists predominantly of drug-naive patients, representing a sample that is slightly different from those studied in the other works, which, having initiated pharmacological treatment, could have some of the biomarkers used in the AL index altered.

## 5 Conclusions

Given the impact of stress, as measured by the AL index, across the psychosis spectrum, it emerges as a valuable tool for both clinical practice and research. It can aid in identifying prognostic factors associated with cardio-metabolic comorbidities and premature mortality while also serving as a potential biomarker to differentiate individuals with varying levels of stress exposure or as a mediator of structural deterioration observed in imaging studies. This meta-analysis is the first to evaluate allostatic load in individuals with schizophrenia spectrum disorders, revealing a clear increase in AL index among individuals with psychosis compared to healthy controls, with those in chronic stages exhibiting even higher levels than those experiencing a first episode. However, due to the limited number of studies, further research is needed to establish a relationship between AL index and symptom severity. Additionally, developing a standardized methodology for measuring AL index is crucial to ensuring more consistent findings, ultimately strengthening the evidence on the mechanisms linking stress and psychosis.

## Data Availability

The original contributions presented in the study are included in the article/**Supplementary Material**. Further inquiries can be directed to the corresponding author.
