# Correction: Allostatic load index across the psychosis spectrum: a systematic review and meta-analysis

**DOI:** 10.3389/fpsyt.2025.1673042

**Published:** 2025-08-08

**Authors:** Lander Madaria, Claudia Aymerich, Borja Pedruzo, Gonzalo Salazar de Pablo, Daniel Alonso-Alconada, Paolo Fusar-Poli, Miguel Ángel Gonzalez-Torres, Ana Catalan

**Affiliations:** ^1^ Psychiatry Department, Basurto University Hospital, Bilbao, Spain; ^2^ Biobizkaia Health Research Institute, Organización Sanitaria Integrada (OSI) Bilbao-Basurto, Bilbao, Spain; ^3^ Neuroscience Department, University of the Basque Country, UPV/EHU, Leioa, Spain; ^4^ Centro de Investigación en Red de Salud Mental (CIBERSAM), Madrid, Spain; ^5^ Department of Child and Adolescent Psychiatry, Institute of Psychiatry, Psychology & Neuroscience (IoPPN), King’s College London, London, United Kingdom; ^6^ Child and Adolescent Mental Health Services, South London and Maudsley NHS Foundation Trust, London, United Kingdom; ^7^ Department of Child and Adolescent Psychiatry, Institute of Psychiatry and Mental Health. Hospital General Universitario Gregorio Marañón School of Medicine, Universidad Complutense, IiSGM, CIBERSAM, Madrid, Spain; ^8^ Department of Cell Biology and Histology, School of Medicine and Nursing, University of the Basque Country (UPV/EHU), Leioa, Spain; ^9^ Early Psychosis – Interventions and Clinical-detection (EPIC) Lab, Department of Psychosis Studies, Institute of Psychiatry, Psychology and Neuroscience, King’s College London, London, United Kingdom; ^10^ Department of Brain and Behavioral Sciences, University of Pavia, Pavia, Italy. Outreach and Support in South-London (OASIS) Service, South London and Maudsley (SLaM) NHS Foundation Trust, London, United Kingdom; ^11^ Department of Psychiatry and Psychotherapy, University Hospital, Ludwig-Maximilian-University (LMU), Munich, Germany

**Keywords:** allostatic load, psychosis, schizophrenia spectrum disorders, first-episode psychosis, allostatic load index

In the published article, there was an error in the **Funding** statement. The organization “Biobizkaia Health Research Institute – OSI Bilbao Basurto Research Commission” was incorrectly named. The **Funding** statement originally read:

“The author(s) declare that financial support was received for the research and/or publication of this article. PFP is supported by #NEXTGENERATIONEU (NGEU), funded by the Ministry of University and Research (MUR), National Recovery and Resilience Plan (NRRP), project MNESYS (PE0000006)—A multiscale integrated approach to the study of the nervous system in health and disease (DN. 1553 11.10.2022). This research received funding from the OSI Bilbao Basurto Research Commission for publication fees.”

This has been corrected to read:

“The author(s) declare that financial support was received for the research and/or publication of this article. PFP is supported by #NEXTGENERATIONEU (NGEU), funded by the Ministry of University and Research (MUR), National Recovery and Resilience Plan (NRRP), project MNESYS (PE0000006) – A multiscale integrated approach to the study of the nervous system in health and disease (DN. 1553 11.10.2022). This research received funding from the Biobizkaia Health Research Institute – OSI Bilbao Basurto Research Commission for publication fees.”

The original version of this article has been updated.

